# The pig as a model for translational research: overview of porcine animal models at Jichi Medical University

**DOI:** 10.1186/2047-1440-1-8

**Published:** 2012-08-16

**Authors:** Eiji Kobayashi, Shuji Hishikawa, Takumi Teratani, Alan T Lefor

**Affiliations:** 1 Center for Development of Advanced Medical Technology, Jichi Medical University, 3311-1, Yakushiji, Shimotsukeshi, Tochigi, 329-0498, Japan; 2 Division of Development Advanced Therapy, Center for Development of Advanced Medical Technology, Jichi Medical University, 3311-1, Yakushiji, Shimotsukeshi, Tochigi, 329-0498, Japan

**Keywords:** Experimental animals, Pig, Translational research

## Abstract

To improve the welfare of experimental animals, investigators seek to respect the 3R principle (Replacement, Reduction, and Refinement). Even when large animal studies are essential before moving to clinical trials, it is important to look for ways to reduce the number of experimental animals used. At the Center for the Development of Advanced Medical Technology, we consider ‘medical’ pigs to be ideal preclinical model systems.

We have been using both wild-type and genetically modified pigs. We began using this approach about 10 years ago with a ‘total pig system’ to model human health and disease for the purposes of both medical skill education and the development of new devices and therapeutic strategies.

At our Center, medical students and residents use pigs to gain experience with surgical skills and train for emergency procedures after appropriate simulation training. Senior clinicians have also used these models to advance the development of innovative tools for endo- and laparoscopic procedures. The Center focuses on translational research for organ transplantation and stem cell therapy. Several pig models have been established for liver, intestine, kidney, pancreas, and lung transplantation. Mesenchymal stromal cells have been established in green fluorescent protein- and red fluorescent protein-transgenic pigs and tested to trans-differentiate organogenesis. A program to establish induced pluripotent stem cells in the pig is ongoing at our Center.

Here, we review our 10 years of activity in this field. Based on our experience in surgical education and research, experimental pigs are valuable models in translational research.

## Introduction

Pigs and humans have anatomical and physiological similarities. First, the immune system of pigs is similar to that of humans, and second, inbred pigs such as Clawn minipigs have genetically defined and fixed major histocompatibility complex, making reproducible studies of immunologic mechanisms possible [1-4]. Therefore, the pig has attracted attention as a valuable preclinical model for medical research. Although there have been few reports of the scientific advantages of using pigs as such models, there are multiple reports of the use of dogs and non-human primates. Because of the ethical limitations on using mongrel dogs for medical education and research in Japan, we decided to use a different medium-sized experimental animal as a model.

From 2001 to 2002, with the help of a grant established by Tochigi Prefecture to promote regional economic activity, we surveyed several institutions in Japan that used pigs as experimental models. Our investigation of the numbers of pigs used in experiments by all of the medical universities in Japan revealed that there was scant information available on the use of pigs. Furthermore, the support system for using these animals as biomedical models was poor. More than 60% of 115 universities surveyed had no experience in the postoperative care of experimental pigs, and fewer than 10 domestic or miniature pigs were used annually at another university. As a result, we started the Tochigi Pig Project to study the use of pigs as biomedical models [5].

### Establishment of a total care system for medical pigs

**Figure 1:**
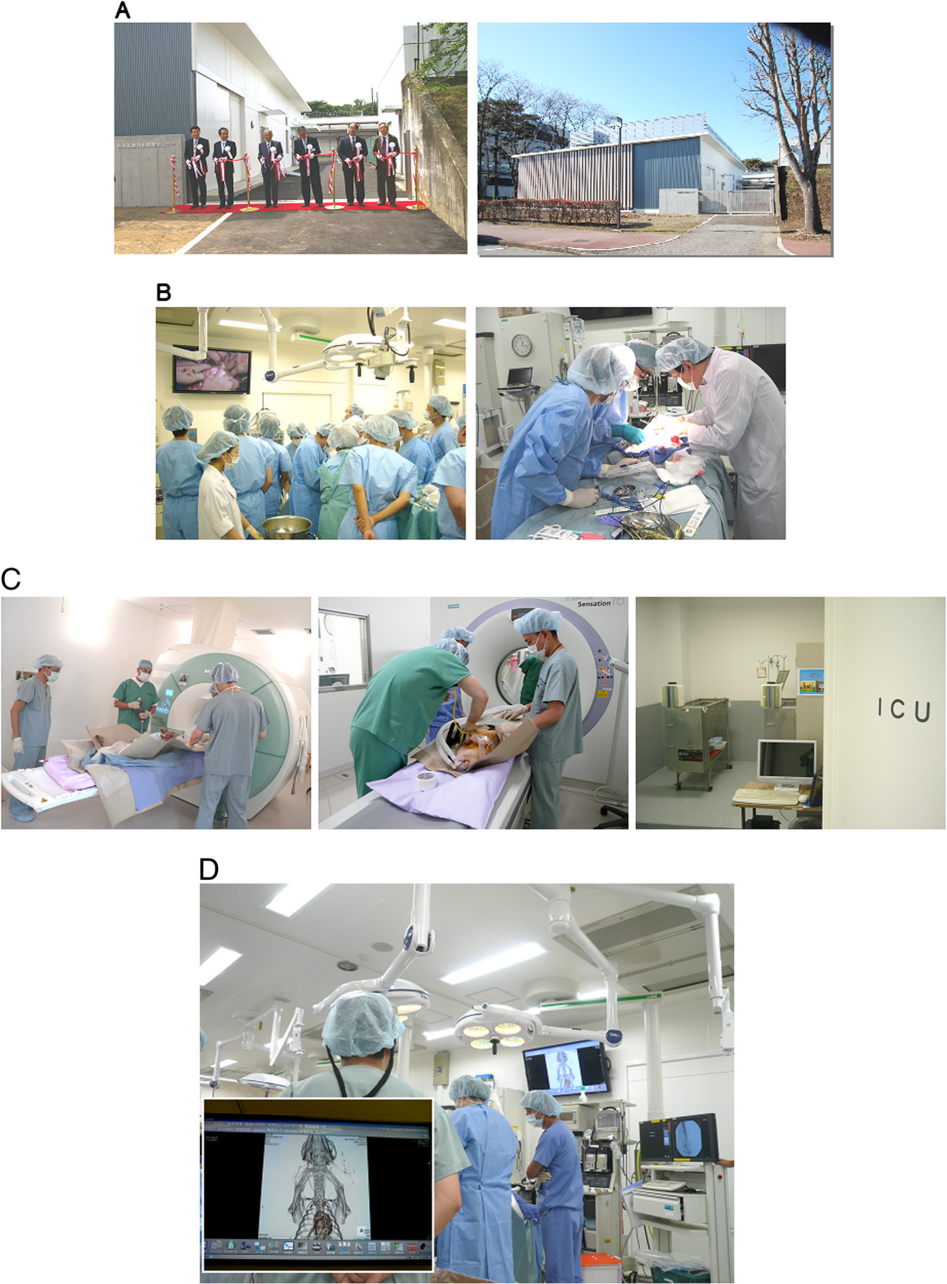
**The CDAMTec research center at Jichi Medical University. A**) Opening ceremony (left panel) and the outside of the pig center (right panel). **B**) Surgical training using pigs. **C**) CDAMTec features MRI (top panel), CT (middle panel), and an ICU (bottom panel). **D**) Surgical simulation system. Inset shows patient data. CDAMTec, Center for Development of Advanced Medical Technology; CT, computed tomography; MRI, magnetic resonance imaging.

Over the last 10 years, we have studied several kinds of pig. They are classified into three categories on the basis of body size. Our new pig research center, the Center for Development of Advanced Medical Technology (CDAMTec), began full-scale operations in 2008 (Figure 1A). CDAMTec has three important goals - education, surgical training, and pre-clinical research - and a number of academic investigators and physicians have been recruited to the center (Figure 1B). CDAMTec provides advanced-level clinical care and testing, including computed tomography (CT), magnetic resonance imaging (MRI), and an ICU (Figure 1C, Additional file 1: Movie S1). Furthermore, an advanced imaging system is used to combine CT and MRI data for surgical support (Figure 1D, Additional file 2: Movies S2 and Additional file 3: Movies S3). The Center is used for surgical training and education in high-risk techniques or techniques where high skill levels are needed, or before the initiation of clinical trials [6].

Jichi Medical University introduced the use of pigs as part of the Bedside Learning phase of clinical surgical education at CDAMTec. The curriculum is composed of four sections: Bioethics, Orientation, Practical training, and Verification of surgical skills.

The Advanced Trauma Operative Management course was established in the United States in 2000 to standardize the operative management of trauma patients. The course is given to senior surgeons and senior surgical residents, by using a one-to-one instruction system. The course was brought to Japan in 2008 and began at CDAMTec. We also offer separate training for surgical residents to improve their skills with a dry-lab and microsurgical training [See Additional file 4: Movie S4].

In addition to training in specific procedures, all users of the facility are educated regarding the welfare of experimental animals. The design of the programs conducted has reduced the overall number of experimental pigs used in training [7].

### Research using domestic pigs

**Figure 2:**
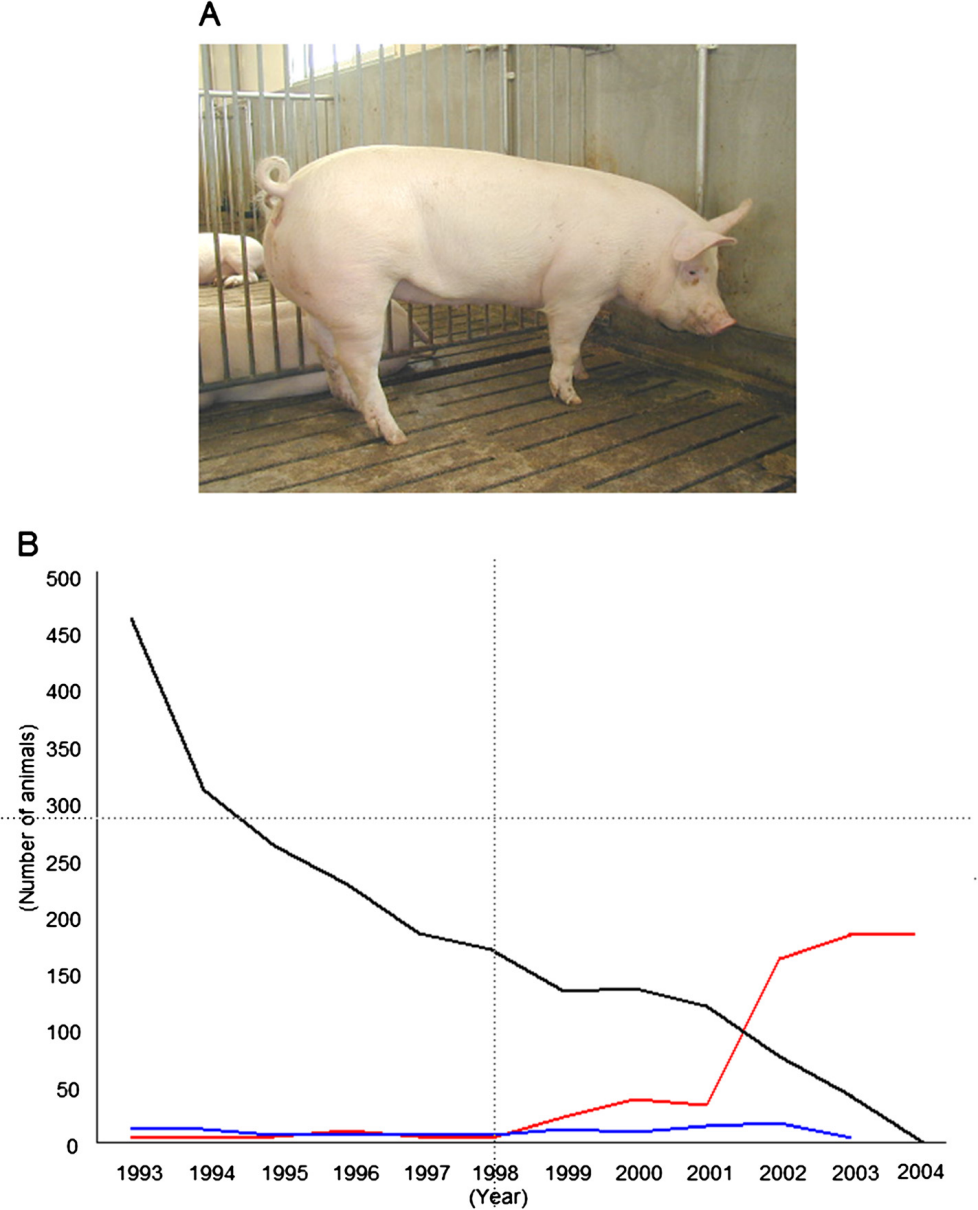
**Changes in use of large animals at Jichi Medical University. A**) Domestic pig. **B**) Numbers of large animals used at our university for research. Pig (red), dog (black), and monkey (blue).

The animals used in these training programs are domestic pigs with a mature body weight of approximately 100 kg. In the early stage of the project, we focused on the medical use of domestic pigs with help from farmers close to the university. Domestic pigs are easily obtained and inexpensive, because they are well established as a food source. They are produced in a three-breed terminal system by crossbreeding among Landrace, Large White, and Duroc colonies (Figure 2A). Young pigs weighing approximately 30 to 40 kg are often used for medical training and research.

At Jichi Medical University before this project was started, medical students practiced their surgical skills on mongrel dogs. Because of recent changes to both animal welfare and public opinion in Japan, mongrel dogs are no longer used for medical education and research. In 2000, the use of all experimental animals in educational courses at Jichi Medical University was changed from dogs to pigs (Figure 2B).

At the same time, studies were initiated to investigate differences in liver metabolism between humans and pigs. A simple surgical model was established that utilizes an internal shunt. First, changes in microsomal P450 isoforms after therapeutic liver resection were studied by using this model [8]. Partial occlusion (portal vein and hepatic artery occlusion) decreased the activities of CYP2C, CYP2E, and CYP3A, but not those of CYP1A and CYP2D. CYP3A, which accounts for an average of 30% of the total P450 content in the human liver, was most susceptible to warm ischemia. The metabolism of the anesthetic drug, propofol, was then examined [9]. Propofol is known to have intra- and extrahepatic metabolic pathways, but the effect of its continuous infusion during long-term anhepatic states had not been determined. Hemodynamic parameters related to the pharmacokinetics of continuously infused propofol (6 mg/kg/hr) were also investigated. Although there were changes in the heart rate, no significant changes in the concentration of hemoglobin or in hemodynamic parameters were observed during the anhepatic phase when propofol was continuously infused. Mixed venous, arterial, and portal vein propofol concentrations were stable during the anhepatic phase.

Using this model, three kinds of infusion solutions were tested, namely lactated Ringer’s solution (LR, Lactec), acetated Ringer’s solution (AR, VeenF), and acetated Ringer’s solution with 1% glucose (AR-G, Phisio140) [10]. Although no major difference was observed in the hemodynamic parameters, arterial blood gas data, or electrolyte concentrations among the three groups, a significant and progressive elevation of lactic acid levels was observed in the LR group. Severe hypoglycemia was found in the LR and AR groups, whereas the AR-G group maintained its blood glucose levels throughout the anhepatic phase.

Hepatitis E virus (HEV) is highly prevalent among domestic pigs, and substantial attention has been given to keeping our center free from this virus [11]. We investigated the prevalence of IgG class antibodies to pig HEV (anti-HEV) and of HEV RNA among 152 domestic pigs at 2 months of age and 38 miniature pigs from 4 to 10 months of age; the pigs had been obtained for research purposes from five farms in Japan. HEV RNA was detected in 38% of the domestic pigs; the 22 HEV isolates recovered from the viremic pigs were 89.8% to 100% identical in the 412-nucleotide sequence of open reading frame 2 and segregated into three clusters within genotype 3. In contrast, all of the miniature pigs, which came from farms different from those where the domestic pigs were sourced, were negative for both anti-HEV and HEV RNA. We concluded that it is important to check that pigs provided for research are HEV free.

We also began a program to share animal tissues, whereby researchers can reuse various organs obtained from euthanized animals [12]. In 2003, the number of pigs used as experimental animals at our center rapidly increased to more than 170. Moreover, the number of chronic experiments has increased; such experiments accounted for 48% of experiments using pigs in 2004. Improvement of the effective utilization of these animals is required from both an economic and an ethical point of view. Experimental pigs undergo secondary use after being euthanized, thus reducing the total number of experimental animals needed for medical research. Overall numbers are similarly reduced through sharing and reuse of miniature-pig tissues and cells for research. We recommend this system, because it improves the quality of medical education and research and facilitates the effective use of tissues and cells through sharing and reuse among different investigators.

Development of an efficient system of pre-culture of pig islets was ideal [13]. This program has continued, resulting in a reduction in the total number of experimental animals used.

### Research using miniature pigs

Although miniature pigs are generally easier to handle and more suitable for medical research than are domestic pigs, miniature pigs are more expensive because of limited annual production for experimental use in Japan. It is important to note that a mature miniature pig weighs 40 to 50 kg; this weight is equivalent to that of an immature domestic pig (Figure 2A).

Platelets promote tissue repair and liver regeneration. After observing a positive response to human thrombopoietin (TPO) in mature miniature pigs, we found that platelets prevent acute liver damage after extended hepatectomy in pigs [14]. Thrombocytosis was induced by the following two methods, and an 80% hepatectomy was performed. In the first method, pigs received thrombopoietin (TPO (+) group), and were compared with a control group (TPO (−) group). In the second method, an experimental group underwent splenectomy (Sp (+) group) to induce reactive thrombocytosis and was compared with a control group (Sp (−) group). Serum concentrations of the enzyme induced by liver damage were significantly lower in the thrombocytotic groups than in the control groups in the early period after hepatectomy. Histopathological examination revealed hemorrhagic necrosis with a bile plug in the control groups, but this phenomenon was not observed in the thrombocytotic groups. We concluded that mature miniature pigs respond to human TPO and that an increased platelet count prevents acute liver damage after extended hepatectomy in this species.

**Figure 3:**
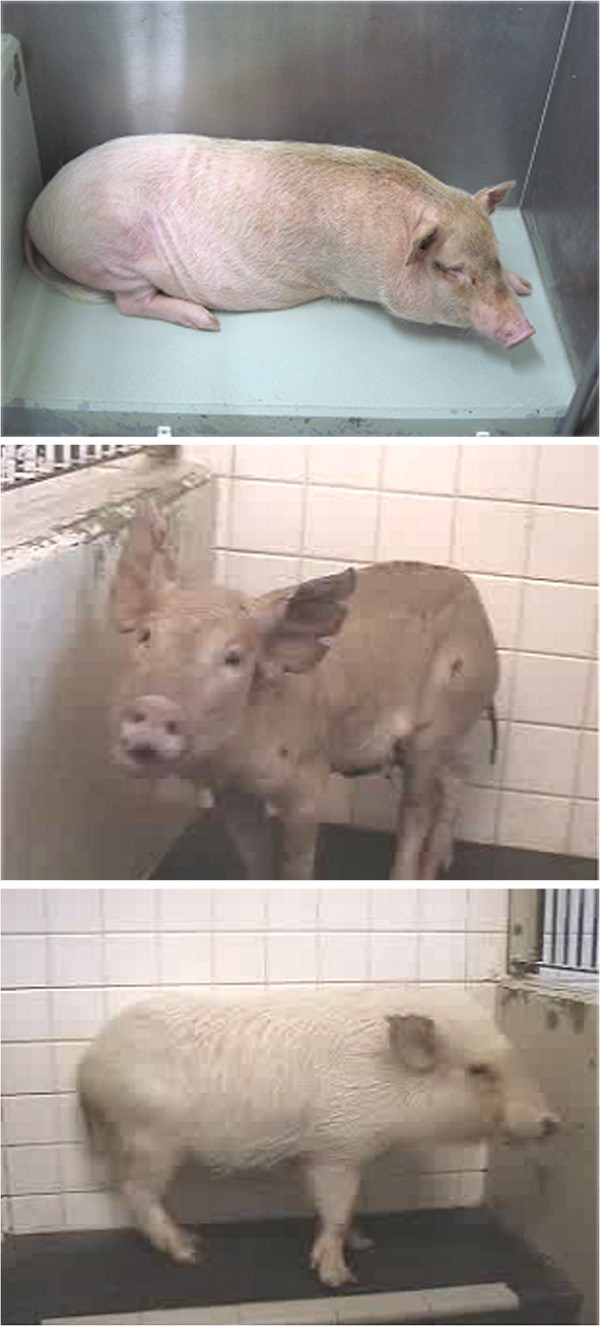
**A variety of miniture pig strains are used at Jichi Medical University for experimental research.** KCG pig (top panel), Mexican hairless pig (middle panel), and Clawn pig (bottom panel).

Investigators must optimize the perioperative care of experimental animals, but little is known about the effects of anesthesia and surgery on serum chemistry in KCG miniature pigs [15] (Figure 3, top panel). Our objective in another study was to examine the influence of fasting and surgery under general anesthesia on 27 serum chemistry parameters in KCG miniature pigs so as to improve preoperative management. Crossbred KCG miniature pigs were used at a mean of 12.3 months of age (range, 8.6 to 14.9 months) and 33.4 kg body weight (range, 24.0 to 40.2 kg). Serum chemistry was evaluated at the beginning and end of a 24-hour fasting period (n = 6). No significant differences were observed between the values tested at the two time points. Partial hemilaminectomy of the lumbar spine was performed in two groups of animals. Those given sevoflurane anesthesia (n = 7) had significant decreases in serum albumin, potassium, inorganic phosphorus, gamma-glutamyltransferase peptidase, cholinesterase, and glucose after surgery compared with levels before surgery. Animals given isoflurane (n = 7) anesthesia had significantly decreased total protein, albumin, triglyceride, phospholipids, sodium, potassium, calcium, alanine aminotransferase, alkaline phosphatase, and glucose after surgery compared with levels before surgery. In a separate experiment (n = 7), serum glucose and insulin also decreased during the postoperative period after isoflurane anesthesia. These results demonstrated that specific serum electrolytes, glucose, and insulin were altered in KCG miniature pigs undergoing general anesthesia. Investigators must be aware of the effects of anesthetic agents on experimental animals so as to provide optimum care and correct the interpretation of experimental data.

Mexican hairless pigs (Figure 3, middle panel), which were developed by Japan’s National Agriculture and Food Research Organization, are highly suited for the evaluation of topical agents, because the structure of their skin is similar to that of humans [16]. To evaluate the pharmacokinetics of topical drugs, *in vitro* permeation studies are performed by using the skin of euthanized animals or human tissues resected at surgery; however, these methods have limitations for evaluating *in vivo* pharmacokinetics. Therefore, we studied the use of Mexican hairless pigs for *in vivo* pharmacokinetics, especially for the evaluation of drug concentrations in tissues. A ketoprofen patch was applied to the backs of Mexican hairless pigs for 24 hours; this was followed by sequential collections of blood from 0 to 36 hours. Skin, subcutaneous fat, fascia, and muscle from the center of the application site were excised 12 hours after patch application. Ketoprofen was first detected in the plasma at 8 hours. The concentration increased until 24 hours and began to decrease after removal of the ketoprofen patch. Ketoprofen concentrations in the tissues decreased with increasing tissue depth, but the amount in the deep muscles, being the lowest among the tissues examined, was still higher than that in the plasma. Drug concentrations are difficult to test in human tissues, and the Mexican hairless pig model appears to be attractive for *in vivo* pharmacokinetic studies of topically applied ketoprofen. This breed has also been used to develop a new medical device to connect the intestinal lumen percutaneously by using a double-balloon method [17].

Clawn miniature pigs (Figure 3, bottom panel) commercially provided by the Japan Farm Clawn Institute are recommended for use in transplantation research, because their SLAs (swine leukocyte antigens) have been fixed, and definite immunological reactions are observed in models of intestinal [18] and lung transplantation [19]. The outcome of highly immunogenic transplantation remains unsatisfactory, despite the development of potent immunosuppressants. There is emerging clinical evidence that, paradoxically, expression of forkhead box P3 (FOXP3, a specific marker for regulatory T cells) is upregulated in graft rejection. Levels of mRNA expression of FOXP3, perforin, Fas-ligand (Fas-L), and interferon gamma-induced protein 10 (IL-10) were quantified in the peripheral blood and reached their highest value as early as postoperative day four, followed by a decline. Increased FOXP3 expression was not observed in recipients given high-dose tacrolimus. In a miniature, such as Clawn pig, transplantation model, FOXP3 mRNA levels in the peripheral blood were upregulated in the early phase of rejection. Thanks to the merits of this inbred colony, induced pluripotent stem (iPS) cells have been established for development by Hanazono [20]. The established iPS cells, which have been confirmed to form teratomas in severe combined immunodeficiency (SCID) mice, are now being tested in a syngeneic combination of mature Clawn pigs.

### Research using genetically modified miniature pigs

As genetic modification techniques have developed, somatic cloning technology has become well established in pigs. Recently, we produced cloned miniature pigs by using oocytes derived from domestic pig ovaries [21]. For the production of viable somatic-cell nuclear transferred (SCNT) miniature pig embryos, the *in vitro* conditions for controlling the quality of recipient oocytes derived from domestic pig ovaries need to be evaluated. To obtain information on the optimum *in vitro* maturation (IVM) conditions for oocytes, we investigated the effect of IVM duration of recipient oocytes on the subsequent development of SCNT miniature pig embryos. We also investigated maturation-promoting factor (MPF) activity in recipient oocytes before and after SCNT, along with the occurrence of premature chromosome condensation (PCC) and the spindle morphologies of donor nuclei following SCNT. The optimal window for the IVM period in terms of the *in vitro* developmental ability of SCNT embryos was 36 to 40 hours after the start of IVM. Use of recipient oocytes matured for 36 or 40 hours, but not 44 or 52 hours, resulted in a high level of MPF activity before and after SCNT and increased the occurrence of PCC in transferred nuclei. The proportion of abnormal spindle-like structures increased with prolongation of the IVM period. In addition, SCNT embryos constructed from recipient cytoplasts obtained after 40 hours of maturation by using fetal fibroblasts of miniature pigs were transferred to surrogate miniature pigs and developed to full term. These results suggest that recipient oocytes matured for 36 hours or 40 hours show effective induction of PCC with a normal cytoskeletal structure because of a high level of MPF activity. Furthermore, the 40 hour IVM period improves the *in vitro* development of SCNT embryos to the blastocyst stage, resulting in the production of viable cloned miniature pigs.

Animal imaging sources have become indispensable in the biological sciences. Specifically, gene-encoded biological probes serve as stable and high-performance tools for visualizing the fate of cells in living animals. Green fluorescent protein (GFP)-transgenic Jinhua pigs have been established and show normal growth and reasonable reproductive activity [22]. In this transgenic pig model, a green emission is observed in the body under an excitation light. We use a somatic cell cloning technique to create new miniature-sized GFP-expressing Jinhua pigs; this enables us to characterize expression profiles in various tissues or organs and under various *ex vivo* culture conditions. Strong GFP expression is observed in the skeletal muscle, pancreas, heart, and kidney. Bone-marrow-derived mesenchymal stromal cells (MSCs), hepatocytes, and islet cells of the pancreas also show detectable expression, with a unique pattern. Moreover, the cloned pigs demonstrate normal growth and fertility, and the introduced GFP gene is stably transmitted to subsequent generations. These GFP-expressing Jinhua pigs can be used as new cellular or tissue light resources for biological imaging in preclinical research fields such as tissue engineering, experimental regenerative medicine, and transplantation. These pigs are also a useful source for imaging in the field of stem cell research. A clinical protocol for intra-articular injections into massive meniscal defects has been tested by using synovial stem cells obtained from GFP-transgenic animals [23].

### Research using micromini pigs

**Figure 4:**
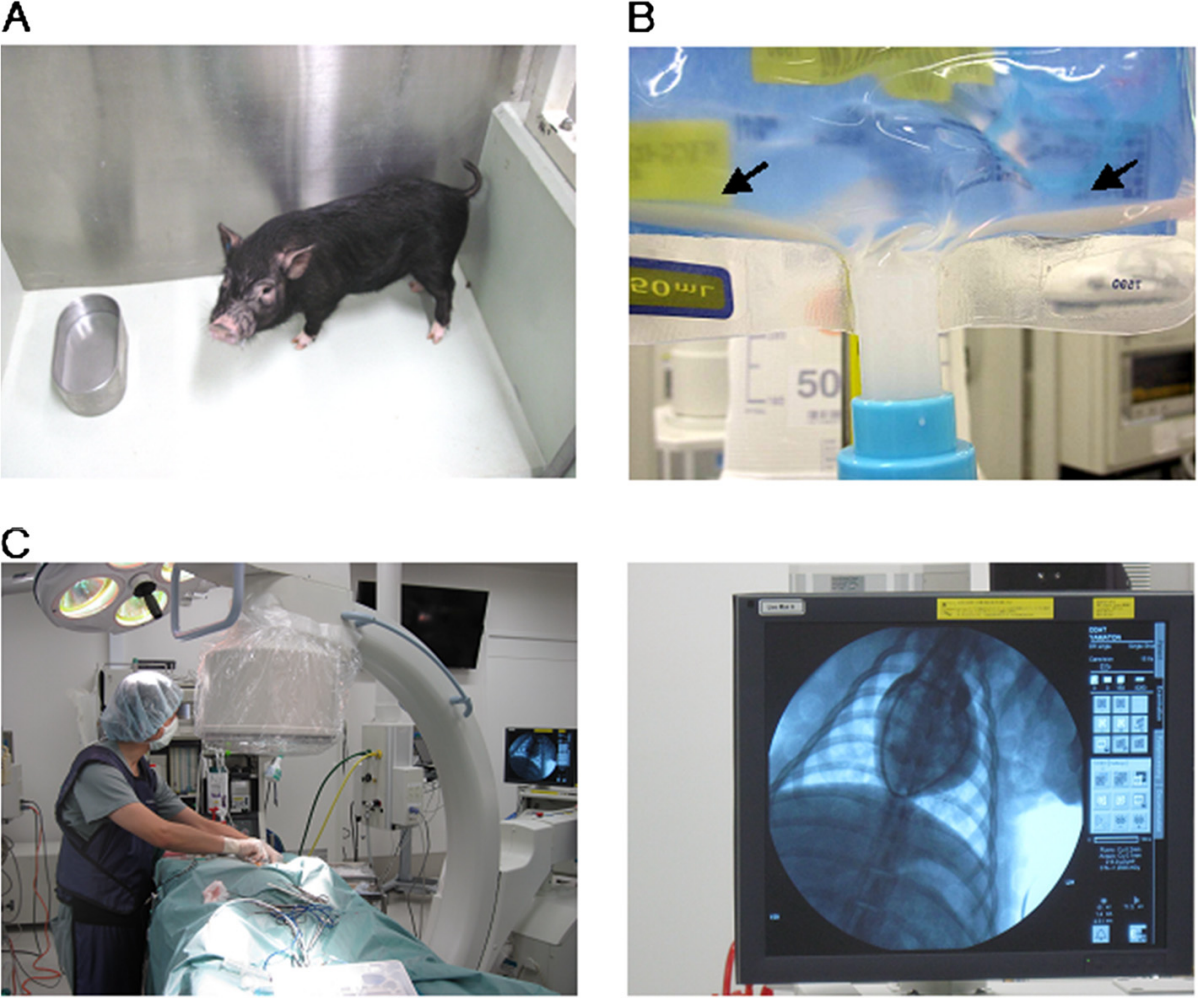
**Cell transplantation models in the pig. A**) Micromini pig. **B**) Cell mixture solution in an infusion pack after one hour. Arrow shows precipitation of cells. **C**) Placement of a pulmonary artery catheter by using a C-arm (left panel) and monitor (right panel).

A very small pig, termed a ‘micromini pig’ is likley to prove suitable for preclinical safety evaluations of new agents (Figure 4A). Although several years might be needed to perform comparative studies of drug toxicology among dogs, monkeys, and pigs, we believe that these micromini pigs will be suitable for use in studies of advanced medical treatments. We have great interest in regenerative medicine and are focusing on the micromini pig model to test its benefits and drawbacks in this field. Because of their moderate body size, micromini pigs are suitable for testing systemic injections of MSCs. This is not the case in rodents, which are too small, and miniature pigs, for which more time is required for stem cell expansion *in vitro*. MSCs are expected to have tissue-regenerative, immunomodulatory, and anti-inflammatory effects [24].

However, a major risk of MSC therapy is pulmonary embolism in the very early phases of treatment. It is important to note that MSCs suspended in saline or culture media easily become sedimentary and aggregate in less than one hour (Figure 4B); this might be related to the risk of embolism-related complications after intravenous injection. A preclinical study using the micromini pig clearly showed that systemic injection of MSCs (1 × 10^7^cells/kg, about 3.0 × 10^8^cells/pig) immersed in normal saline without heparin (200 mL, infusion time 40 minutes) caused the pulmonary arterial pressure to increase by more than 30 mm Hg, as determined by monitoring with a pulmonary artery catheter (Figure 4C). However, by using a solution that we developed in a liver ischemia–reperfusion model in pigs, we found that allogenic MSC transplantation had a therapeutic effect (manuscript in preparation). Additionally, for the purpose of these procedures, the pig’s anatomy is suffiicently similar to that of humans for X-ray to be useful in monitoring blood flow to the liver [See Additional file 5: Movie S5]. Thus, the micromini pig was suitable for the development of an MSC-aggregation inhibition solution and is useful for evaluating treatment effects and creating a surgical model that mimics the human one.

## Conclusions

We have described our 10 years of experience with the development and use of several porcine models as biomedical research tools. Development of disease models in pigs is essential for further expanding the use of pigs in effective preclinical experiments, because few spontaneous disease models are available in pigs, unlike in dogs. Recent advances in genetic technology in pigs have shown that these experimental animals are now suitable, mature biomedical models.

## Competing interests

EK has been a visiting professor at CDAMTec and a special advisor to Otsuka Pharmaceutical Factory Inc. (Naruto, Japan) from 2009. There are no patents, products in development, or marketed products to declare. The position held by EK does not alter the authors adherence to all of the Transplantation Research policies on sharing data and materials, as described in detail online in the Guide for Authors. The other authors declare no competing financial interests.

## Authors’ contributions

EK designed and coordinated the project. SH and AL performed educational aspects of the project. TT conducted the research project. All authors read and approved the final manuscript.

## Supplementary Material

Additional file 1 **Movie S1.** CT and MRI dedicated pig in CDAMTec. Click here for file

Additional file 2 **Movie S2.** Surgical training by an advanced imaging system. Click here for file

Additional file 3 **Movie S3.** Demonstration of CT and MRI imaging analysis data on PC monitor. Click here for file

Additional file 4 **Movie S4.** The Advanced Trauma Operative Management course. Click here for file

Additional file 5 **Movie S5.** Mobile X-ray system as C-arm. Click here for file
